# Integrated Analysis of *CRY1* Gene Expression and InDel Polymorphism Reveals Associations with Ovarian Morphological Traits in Chinese Holstein Dairy Cattle

**DOI:** 10.3390/biom16081201

**Published:** 2026-08-17

**Authors:** Xuanbo Chen, Enhui Jiang, Yuta Yang, Haotian Zhang, Zhaoyu Liu, Ebadu Areb, Yongsheng Wang, Xianyong Lan

**Affiliations:** 1 Key Laboratory of Animal Genetics, Breeding and Reproduction of Shaanxi Province, College of Animal Science and Technology, Northwest A&F University, Yangling 712100, China; 2023010827@nwafu.edu.cn (X.C.); jiangenhui666@nwafu.edu.cn (E.J.); yydahsy@163.com (Y.Y.); zht0501@nwafu.edu.cn (H.Z.); liuzhaoyu19939469829@nwafu.edu.cn (Z.L.); ebaduareb3@nwafu.edu.cn (E.A.); 2 College of Veterinary Medicine, Northwest A&F University, Yangling 712100, China

**Keywords:** *CRY1*, circadian rhythm, Chinese Holstein, gene expression, InDel polymorphism, ovarian traits, molecular marker

## Abstract

Clock genes, such as cryptochrome 1 (*CRY1*), exhibit rhythmic expression in the reproductive organs. This gene is a key component of the circadian clock and is involved in various physiological processes, including reproduction, suggesting that it may be linked to ovarian activity via neuroendocrine pathways. However, the association between *CRY1* variants and ovarian traits in dairy cows is unclear. In this study we used qRT-PCR to assay the mRNA expression of this gene, and found that *CRY1* expression was highest in oocytes in several tissues; subsequently, we found that *CRY1* expression was highest in the ovary in Chinese Holstein dairy cattle, followed by muscle and spleen tissues (*p* < 0.01). Next, we identified a six bp insertion/deletion (indel) locus within the *CRY1* gene in Chinese Holstein dairy cattle. Only two genotypes were detected: II (*n* = 854, 83.7%) and ID (*n* = 166, 16.3%). Association analysis revealed that the identified indel was significantly associated with dominant follicle and corpus albicans diameters (*p* < 0.05) during metestrus. Specifically, individuals with the ID genotype exhibited smaller dominant follicles and corpus albicans than those with the II genotype. Our preliminary findings suggest that *CRY1* is associated with specific ovarian morphological traits and may serve as a basis for further investigation of its potential role in bovine reproductive function.

## 1. Introduction

Simultaneously improving milk production and fertility is challenging in dairy production. Although milk production has greatly improved [1], the reproductive efficiency of cows has decreased in the past few decades [2]. Consequently, improving fertility has become an urgent priority in recent years. The determinants of dairy cow fertility are numerous and complex [2]. As a crucial component of the female reproductive system, the ovary not only generates gametes but also functions as an endocrine gland that provides fertility-associated steroid hormones [2]. Therefore, it has long been a subject of exploration in the field of female reproduction [3]. Cows with larger ovaries tend to have a higher number of follicles and oocyte masses [4].

The *CRY1* protein regulates cell metabolism and proliferation and is involved in tumorigenesis. It plays an important role at several levels in mammals by controlling circadian rhythms [5]. Disruption of the circadian clock can cause diseases of a wide range of systems and organs [6,7], such as pulmonary diseases [8], thyroid cancer [9], and infertility [10]. The clock can be affected by many factors [11]; among them, genetic factors are the key to the molecular structure of the circadian clock, and clock genes and proteins form an intricate regulatory network for the clock [12,13]. Cryptochromes play a pivotal role in circadian clock maintenance [14] and other physiological processes. The Cryptochrome 1 (*CRY1*) gene regulates cell metabolism and proliferation [15] and is involved in tumorigenesis [16]. For example, circadian behaviors are disrupted in mice that are homozygous null for *CRY1* and *CRY2* [17]. Female mice deficient in *CRY1* or *CRY2* exhibit irregular and unstable estrous cycles and infertility in middle age [18], indicating that cryptochrome genes may affect female reproductive functions. Furthermore, other evidence suggests a profound bidirectional link between circadian rhythms and reproductive functions [19,20]. The circadian timing system modulates diverse physiological processes via intricate hormonal and neural pathways; consequently, dysregulation of clock gene expression can precipitate a spectrum of reproductive impairments [21] and metabolic shifts [22]. In livestock, this molecular oscillatory machinery is intimately integrated with the hypothalamic–pituitary–ovarian (HPO) axis, which orchestrates the rhythmic secretion of gonadotropins essential for follicular recruitment and ovulation [23]. Given the intensive metabolic demands placed on high-yield dairy cows [24], *CRY1* may function as a pivotal metabolic–reproductive sensor bridging systemic energy homeostasis with localized ovarian function.

Studies have shown that clock genes have the potential to influence the fertility of female livestock, which may help solve the problem of fertility decline in cows [25]. According to Huang et al. (2022) [26], cryptochrome genes affect the litter size of sheep, which preliminarily confirmed the association between clock genes and fertility. A recent study investigating the function of *CRY1* using single-nucleotide polymorphisms within the gene demonstrated the practicability of employing variants to further investigate clock genes. It has been reported that clock genes such as *BMAL1*, *CLOCK*, and *CRY1* exhibit rhythmic expression in the ovary [27], indicating that there is an association between clock genes and ovary function. However, the precise relationship between the ovary and clock genes is unclear. In this study, we investigated the polymorphisms of the bovine *CRY1* gene to clarify the association between the *CRY1* gene and the ovary. To investigate genetic variants and associations with ovarian traits, data were collected from 1020 Chinese Holstein dairy cattle at different stages of the estrous cycle. This study provides a preliminary potential marker for breeding improvement programs.

However, the relationship between the ovary and clock genes remains unclear. Therefore, the aim of this study was to conduct a preliminary investigation into tissue-specific CRY1 expression, as well as to identify functional InDel polymorphisms and evaluate their nominal associations with ovarian morphological traits in Chinese Holstein cattle. This investigation would generate hypotheses and provide foundational data to determine whether CRY1 represents a potential candidate for further exploration of its role in bovine reproductive function.

## 2. Materials and Methods

### 2.1. Ethics Approval

All experimental procedures involving animals were conducted in strict accordance with the relevant guidelines and regulations and were approved by the Institutional Animal Care and Use Committee of Northwest A&F University (IACUC-NWAFU)) (protocol code DK20220201 and date of approval 1 February 2022).

### 2.2. Sample Collection and Morphological Classification of Ovarian Tissues Across Estrous Cycle Stages

Ovary samples were obtained from 1020 Chinese Holstein dairy cows at a commercial abattoir in Xi’an, China. All animals were clinically healthy at the time of slaughter, as confirmed by veterinary inspection, with no detectable reproductive tract diseases. The inclusion criteria were as follows: (1) age of 5–6 years and parity of 2–4; (2) non-pregnant status, confirmed by uterine examination and absence of embryos; (3) no history of reproductive disorders; (4) origin from conventional dairy farms with similar management practices, including tie-stall housing, comparable diet formulations, and standardized milking routines; and (5) slaughter at the same abattoir under standard commercial conditions. The primary reasons for culling were low milk production and reproductive failure, defined as failure to conceive after multiple inseminations. All 1020 animals were multiparous cows, and no bulls were included in the study.

Ovarian tissues were classified into different estrous cycle stages based on corpus luteum morphology and follicular characteristics, as described previously [3,28]. Briefly, estrus was defined as the presence of a large dominant follicle and the absence of an active corpus luteum (CL). Proestrus was characterized by the absence of a functional CL and the presence of developing follicles in the ovaries. Metestrus was defined as the presence of early-stage CL, including type I (cone-shaped) and type II (crater-shaped) CL. Diestrus was characterized by the presence of mature CL, including type III (mushroom-shaped) and type IV CL. The corpus albicans, representing regressed CL from previous cycles, was recorded but not used as a primary criterion for estrous cycle classification. In addition, CL color and vascularization (low, moderate, or high) were recorded to assist with morphological evaluation. Each ovary was measured for length, width, height, and weight, and the corpus lutea were evaluated for diameter and number [29]. The distribution of samples across estrous cycle stages was as follows: estrus (*n* = 115), diestrus (*n* = 498), metestrus (*n* = 268), proestrus (*n* = 126), and unclassified (*n* = 13, excluded).

The differing denominators for various traits are due to the biological characteristics of the structures. For instance, not every ovary contains a dominant follicle (DF) or a corpus albicans (CA) at a given stage of the estrous cycle. Only ovaries in which a DF or CA was present and measurable were included in the analysis for those specific traits. Similarly, the corpus luteum (CL) was not always identifiable or measurable in every ovary, particularly during the estrus and proestrus stages where a functional CL is absent. This biological variation explains the differences in sample sizes (*n*) reported for each trait. For example, during the metestrus stage, the number of ovaries with a measurable dominant follicle (*n* = 93) is smaller than the total number of ovaries in that stage (*n* = 268) because a DF is not always present at this specific phase.

For genotype frequency comparison across breeds, DNA samples from Charolais (*n* = 54) and Simmental (*n* = 40) cattle were obtained from previous collaborative studies. Blood samples were collected from the tail vein into EDTA-anticoagulated tubes with informed owner consent. Genomic DNA was extracted from whole blood using the salt-extraction method [30].

### 2.3. DNA Extraction and Total RNA Isolation

After collection, the ovary samples were packed in sample sacks, marked with the number, and stored at −80 °C. In addition, the CL, the dominant follicle (defined as a follicle with a diameter ≥ 8 mm, corresponding to the physiological stage of follicle deviation [31,32]), and the corpus albicans (CA) were identified and quantified. The maximum diameter of each structure was measured as a morphological indicator of ovarian activity. After aspirating the liquor inside the larger follicles, we randomly cut the medulla part from the ovaries for DNA extraction (but avoided cutting the CL and corpus albicans). Genomic DNA extraction was performed following the standard procedures described by Aljanabi and Martínez (1997) [30]. Nanodrop 2000 (Thermo Fisher Scientific, Waltham, MA, USA) was used to quantify and assess of the purity of the extracted DNA samples. DNA samples were diluted to 20 ng/µL and preserved at −40 °C.

For RNA extraction, additional tissues (spleen, lung, kidney, muscle, brain, and ovary) were collected from six healthy multiparous Holstein cows (5–6 years old) at the same abattoir. Tissues were collected within 30 min post-slaughter, immediately frozen in liquid nitrogen, and stored at −80 °C. Total RNA was extracted using RNAex Pro Reagent (Accurate Biology, Hunan, China) according to the manufacturer’s instructions. RNA concentration and purity were assessed using a NanoDrop spectrophotometer (Thermo Fisher Scientific, Waltham, MA, USA) [33]. Only samples with A260/A280 ratios between 1.8 and 2.0 were used for subsequent analyses. Genomic DNA was removed using DNase I (Thermo Fisher Scientific, Waltham, MA, USA) treatment according to the manufacturer’s protocol. No-reverse-transcriptase (NRT) controls and no-template controls (NTC) were included in all qPCR runs to confirm the absence of genomic DNA contamination and primer dimers, respectively.

### 2.4. cDNA Synthesis and Reverse Transcriptase Quantitative Real-Time PCR (RT-qPCR)

Reverse transcription was performed using the Hifair^®^ III 1st Strand cDNA Synthesis SuperMix for qPCR (YEASEN, Shanghai, China) with 1 μg of total RNA as the template, according to the manufacturer’s protocol. cDNA from the spleen, lung, kidney, muscle, brain, and ovary was used to analyze mRNA expression. The primer for RT-qPCR was designed to span an exon–exon junction and its efficiency was determined using a standard curve generated from a 5-fold serial dilution of pooled cDNA; efficiencies ranged from 90% to 110% for all primers, with R^2^ > 0.99. The RT-qPCR mixture (20 µL) contained 10 µL of 2x SYBR Premix Ex Taq (Takara Biotech, Shiga, Japan), 2 µL of cDNA (1:100 dilution), 7.2 µL distilled water, and 0.4 µL of forward and reverse primers. To increase the efficiency, each cDNA sample was replicated three times. A melt curve analysis was performed to verify the specificity of amplification (ramping from 60 °C to 95 °C at 0.5 °C increments). Ct values showed a standard deviation < 0.25 across technical replicates. Relative gene expression levels were calculated using the 2^−ΔΔCt^ method and normalized to the reference gene (*GAPDH*). To improve accuracy, three replicates were performed for each tissue sample [34].

### 2.5. Primer Design and Polymorphism Detection

Five InDel variants (rs133189221, rs442912813, rs526350742, rs454970402, and rs800313145) over 5 bp in length were selected from the Ensembl database (https://asia.ensembl.org/index.html, accessed on 15 July 2025) for the *CRY1* gene (Table 1). A primer was designed for each variant based on the sequence of the bovine *CRY1* gene (ID: 535947, NC_037332.1:70260216-70356120) using Primer Premier 5 software, and synthesized by Sangon Biotech (Shanghai, China).

### 2.6. Polymerase Chain Reaction Amplification and Genotyping of CRY1

Amplification was performed using a touchdown polymerase chain reaction (PCR) [35]. The touchdown PCR profile consisted of an initial 5 min denaturation at 95 °C, followed by 12 cycles in which the annealing temperature dropped from 68 °C to 50 °C (decreasing 3 °C every two cycles). Each cycle included denaturation at 95 °C (30 s), annealing (30 s), and extension at 72 °C (30 s). The program was concluded after 30 additional cycles using a fixed annealing temperature of 50 °C. The PCR products were electrophoresed on 3.0% agarose gels to identify the genotypes of each individual sample. Genotypes were determined using a DNA marker according to electrophoretic band patterns. A single band migrating farther from the sample well was classified as the DD genotype (Deletion/Deletion), whereas a single band closer to the well was classified as the II genotype (Insertion/Insertion). Samples displaying two bands were classified as having the ID genotype (Insertion/Deletion). Notably, the DD genotype was not observed in this population. The PCR products were sent to Tsingke Biotechnology Company (Beijing, China) for Sanger sequencing.

### 2.7. Tissue Expression Profiling and Relative Expression Analysis of CRY1

Variant information at position 5:70,335,655 was extracted from previously generated whole-genome re-sequencing data from our laboratory. The distribution and allele frequencies of the different genotypes at this locus were calculated. The mRNA expression profile of *CRY1* was analyzed using the publicly available CattleGTEx dataset (Gene_TPM_Matrix.txt.gz) [36]. TPM expression data for *CRY1* (ENSBTAG00000010149) were extracted from all available tissues. In total, expression data from 94 tissues comprising 26,414 samples were collected. For subsequent analyses, only tissues with a sample size ≥ 5 was retained. The expression levels were normalized to the median_log_2_(TPM + 1), and the tissues were ranked accordingly. The top 20 tissues with the highest median expression levels were selected for the visualization. For reproductive-related tissues, three categories, including the female reproductive system, male reproductive system, and embryo, were selected based on the system classification. The expression levels of *CRY1* in these tissues were ranked using median_log_2_(TPM + 1) and presented accordingly.

### 2.8. Statistical Analysis

CattleGTEx public expression data (*n* = 26,414 samples, 94 tissues) was used to generate the tissue expression profile of *CRY1*. Also, analysis of expected heterozygosity (He), observed homozygosity (Ho), effective allele number (Ne), polymorphism information content (PIC), and Hardy–Weinberg equilibrium (HWE) for Chinese Holstein cows (*n* = 1.020) was performed using the SHEsis online platform (http://analysis.bio-x.cn). The association between the variants and ovarian traits was determined using an independent-samples *t*-test with SPSS software (version 25.0). Only variants possessing at least two genotypes with a minimum of three individuals per genotype (*n* > 3) were included in the statistical analysis. Similarly, a comparison of allele frequency was carried out for Charolais (*n* = 54) and Simmental (*n* = 40) breeds.

## 3. Results

### 3.1. Tissue Distribution and Reproductive Expression Profile of CRY1 in Cattle

**Figure 1:**
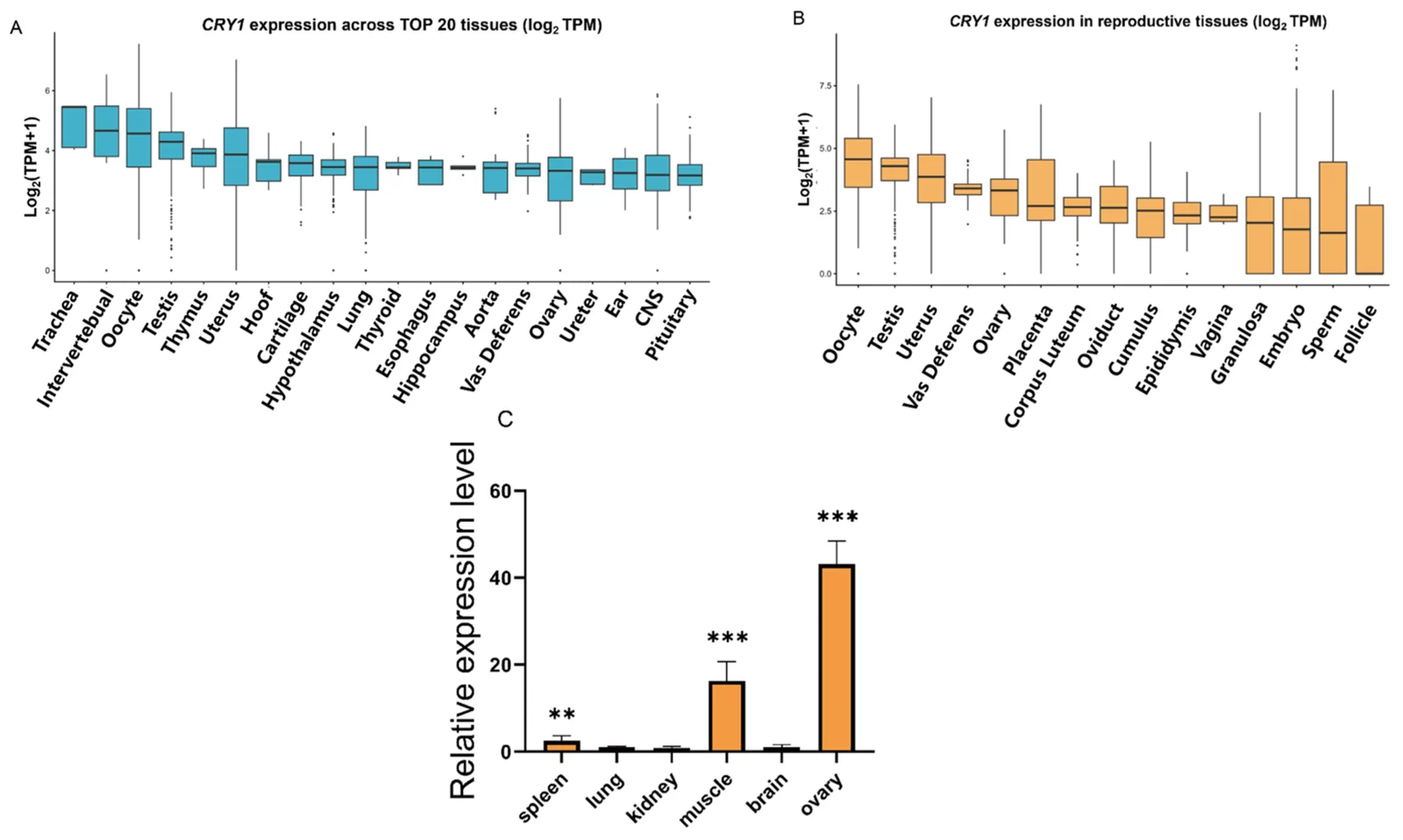
Expression profile of the *CRY1* gene across multiple tissues. (**A**) Boxplot showing *CRY1* expression profile across the top 20 tissues based on log_2_(TPM + 1) values. Higher expression was observed in tissues such as testis, mammary gland and ovary compared with other tissues. (**B**) Expression profile of *CRY1* gene in reproductive-related tissues represented as log_2_(TPM + 1) values. (**C**) Relative expression levels of *CRY1* gene determined by RT-qPCR in selected tissues. The ovary showed the highest expression, followed by muscle and the spleen. Data are presented as mean ± SEM. ** *p* < 0.01, *** *p* < 0.001.

*CRY1* expression was analyzed across 94 tissues comprising 26,414 samples. The top 20 tissues with the highest expression levels are presented in Figure 1A. *CRY1* was highly expressed in the trachea, intervertebral tissue, oocyte, testis, thymus, uterus, hoof, cartilage, hypothalamus, lung, thyroid, esophagus, hippocampus, aorta, vas deferens, ovary, ureter, ear, central nervous system (CNS), and pituitary. Further analysis focused on reproductive-related tissues by selecting three categories from the system classification: female reproductive system, male reproductive system, and embryo. *CRY1* expression was highest in oocytes, followed by the testis, uterus, vas deferens, ovary, placenta, corpus luteum, oviduct, cumulus, epididymis, vagina, granulosa, embryo, sperm, and follicle (Figure 1B).

Subsequently, RT-qPCR analysis was performed using Holstein cattle tissues. *CRY1* expression was highest in the ovary, followed by muscle and spleen tissues (*p* < 0.01, Figure 1C).

### 3.2. Genotyping of the Indel in the Bovine CRY1 Gene

**Figure 2:**
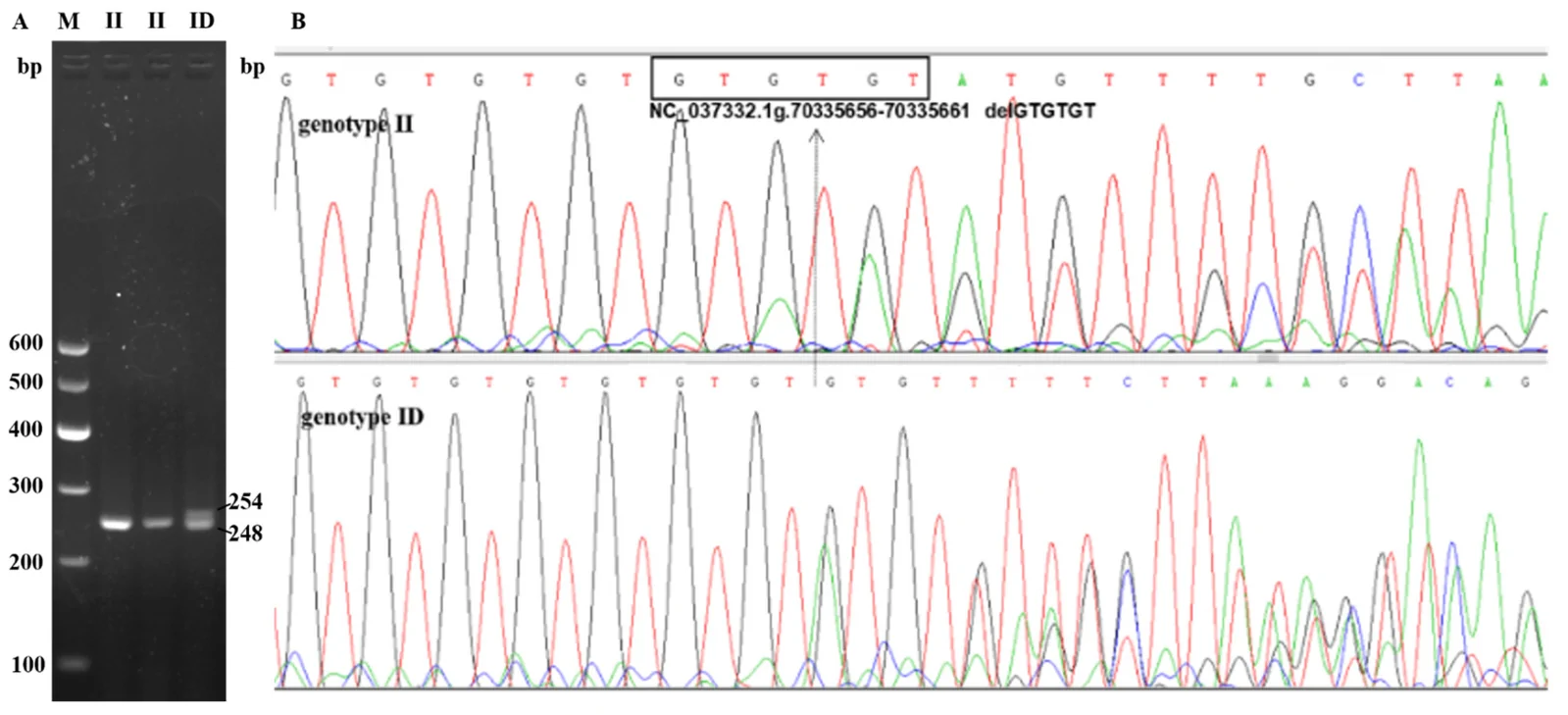
Sequence chroma and electrophoresis pattern of the InDel within *CRY1* gene. (**A**) The electrophoresis pattern of P1-6-bp-del in bovine *CRY1* gene. (**B**) Sanger sequencing diagram of P1-6-bp-del of bovine *CRY1* gene.

Among examined variants, only one mutation site (rs133189221, P1-D6-bp) was confirmed to be polymorphic in the sampled Chinese Holstein dairy cattle. It had two genotypes: the homozygote insertion type (II, 254 bp) and the heterozygote type (ID, 254 and 248 bp) (Figure 2A,B).

### 3.3. Genotypic Frequencies and Population Genetic Parameters

From the genotyping results in Chinese Holstein dairy cattle, the genotypic frequency, allele frequency, and population indices (Ho, He, Ne, and PIC) were analyzed (Table 2). The genotypic frequencies of II (*n* = 854; 0.837) were much higher than those of ID (*n* = 166; 0.163), and the allelic frequencies of I and D were 0.919 and 0.081, respectively. Furthermore, based on the selected loci, the population deviated from HWE (*p* < 0.05), which may be related to mutation, selection, and other factors. The population had low genetic polymorphism (PIC < 0.25) (Table 2). Similarly, allele frequency distributions of *CRY1* in different cattle breeds are presented in Table 3. Charolais cattle showed a fixed allele I (frequency = 1.000) with no allele D detected, whereas Simmental cattle exhibited allele frequencies of 0.950 for I and 0.050 for D.

### 3.4. Associations Between Genotypes and Ovarian Traits

Ovarian trait data and genotyping results revealed an association between P1-D6-bp (Table 4 and Table A1). In the metestrus cycle, P1-D6-bp was significantly associated with the diameter of the dominant follicle and corpus albicans (*p* < 0.05). Individuals with the II genotype exhibited dominant follicles and corpus albicans with larger diameters than those with the ID genotype. However, P1-D6-bp showed no association with ovarian traits at the other three estrous cycle stages.

## 4. Discussion

### 4.1. CRY1 Tissue Expression

The circadian clock is thought to modulate fertility by coordinating reproductive processes, such as the estrus cycle and ovulation [19,20]. As a core component of the molecular oscillatory machinery, *CRY1* contributes to the fundamental structure of the circadian timing system [37]. In this study, we observed the expression pattern of *CRY1* in multiple tissues, which is consistent with its role as a core component of the mammalian circadian rhythm system [38]. Previous studies have demonstrated that core clock genes exhibit broad yet tissue-specific expression characteristics in mammals, suggesting the pivotal role of peripheral clocks in regulating tissue-specific physiological metabolism [39,40,41].

Although the central role of *CRY1* in the mammalian SCN is well-established, its expression across different animal taxa displays remarkable diversity and adaptability. In mammalian peripheral tissues, the liver exhibits the highest amplitude of *CRY1* expression, which is independently modulated by feeding rhythms, whereas *CRY1* is abundant but arrhythmic in the reproductive system [42,43]. In birds, *CRY1* is co-expressed in the retina and pineal gland, where it contributes to photoperiodic integration; European robin *CRY1* was long considered a magnetoreceptor, although this view has recently been challenged by structural evidence [44]. In reptiles and amphibians, *CRY1* expression has been linked to temperature-dependent rhythms and metamorphic development, respectively [45]. In fish, genomic duplication has given rise to functionally divergent paralogs; in seasonally breeding species, *CRY1* expression in the hypothalamus–pituitary–gonadal axis correlates positively with GnRH, acting as a transducer of photoperiodic signals [46], whereas in cave-dwelling and deep-sea fish, prolonged absence of light has led to reduced expression and sequence degeneration [47]. Among insects, Drosophila carries a single Cry gene that functions primarily as a photoreceptor for light resetting, while honeybees and silkworms possess *CRY1* and CRY2, with *CRY1* mediating light-dependent behavioral regulation [48,49]. Collectively, these studies indicate that *CRY1* not only plays a central role in time sensing but has also acquired diverse species-specific non-circadian functions in reproduction, development, and navigation. Of particular interest is the abundant expression of *CRY1* in reproductive tissues across multiple species, suggesting a conserved but incompletely understood role in reproductive regulation.

Our observation that *CRY1* is highly expressed in oocytes and reproductive tissues was further validated by RT-qPCR in Chinese Holstein dairy cattle, which confirmed its enrichment in the ovary and was consistent with transcriptomic data. This expression pattern aligns with previous reports that circadian genes are actively expressed throughout the hypothalamic–pituitary–gonadal (HPG) axis and within reproductive organs, directly participating in gametogenesis, ovulation cycles, and early embryonic development [19,50,51]. Together, these findings support the hypothesis that genetic variants in *CRY1* may influence reproductive efficiency. Nevertheless, the specific effects of such polymorphisms on ovarian traits remain to be systematically characterized.

### 4.2. CRY1 in Ovarian Physiology

According to Wiggins and Legge (2016) [52], *CRY1* expression is significant across various follicular stages, including primordial, primary, and antral follicles, as well as the CL. These findings suggest that *CRY1* may be associated with ovarian function through its potential involvement in follicle development. Follicular growth is a complex process modulated by hormones, whereas follicles themselves serve as endocrine units. Previous studies have suggested that the sensitivity of ovarian follicular cells to gonadotropins may be associated with the activity of clock genes [53]. Consequently, endocrine factors could potentially contribute to the observed association between circadian genes and ovarian traits.

The potential association between *CRY1* and ovarian activity may be discussed from neuroendocrine and genetic perspectives. It is established that estradiol can modulate the expression of light-responsive genes and transcription factors in the SCN [54], where the presence of estrogen receptors has been confirmed [55,56,57]. Environmental factors, such as changing photoperiodism, are known to influence the rhythmic expression of clock genes, including *CRY1* [58]. Previous studies have suggested potential associations between ovarian steroids, the SCN, and circadian rhythms, which may be related to ovarian function. In this context, the P1-D6-bp mutation may be associated with an altered response within this neuroendocrine axis, potentially contributing to the observed variations in follicle and corpus albicans size. However, as environmental light conditions were not monitored in this study, the direct impact of photoperiodism remains a subject for further investigation. Disruption of *CRY1* function could therefore alter the timing and amplitude of hormone production, affecting follicular development and luteal function. However, direct evidence linking *CRY1* variants to altered steroidogenesis in cattle is currently lacking and warrants further investigation.

### 4.3. Functional Implications of the P1-D6-bp InDel

The evaluated 6 bp InDel (rs133189221) is located in intron 1 of the bovine *CRY1* gene. While intronic variants are often considered non-functional, they can affect gene expression through several mechanisms. First, intronic sequences may contain enhancer or silencer elements that regulate transcription. The P1-D6-bp InDel could disrupt transcription factor binding sites, thereby altering *CRY1* expression levels. Second, intronic variants can affect splicing regulation by creating or destroying splice enhancers or silencers [59]. Third, intronic regions may influence chromatin accessibility and three-dimensional genome organization, affecting long-range regulatory interactions [60]. The P1-D6-bp mutation might associate with an interrupted regulatory process, which could potentially contribute to the decreased diameter of dominant follicles and corpus albicans observed in this study.

*CRY1* allele I was found to be the dominant allele across all tested populations, reaching fixation (frequency = 1.000) in Charolais cattle and maintaining an exceptionally high frequency (0.919) in Chinese Holsteins. This high degree of conservation suggests that allele I may have been favored during long-term evolutionary processes or through intensive artificial selection for production traits in these breeds. Chinese Holstein dairy cattle exhibited a much higher frequency of the II genotype (0.837) than the ID genotype (0.163). The low genetic diversity of this population was evidenced by a PIC value of less than 0.25.

A notable deviation from Hardy–Weinberg equilibrium was detected at this locus (p<0.05). In contemporary dairy cattle, such deviations are frequently observed and are typically driven by a combination of factors, including intensive selection pressure, non-random mating practices associated with widespread AI sire usage [61,62], and underlying population substructure (e.g., Wahlund effect). For the Chinese Holstein population examined here, the significant HWE imbalance is probably a consequence of cumulative selection for production traits over generations, which can reduce effective population size and cause linked variation to drift. In addition, the heavy reliance on elite sires for artificial insemination inevitably introduces non-random mating, further pushing genotype frequencies away from HWE predictions.

However, due to the lack of genome-wide marker data and detailed pedigree information, these factors could not be formally evaluated in the present study. Our analysis of 1020 Chinese Holstein dairy cattle revealed that the P1-D6-bp variant was significantly associated with the diameter of dominant follicles and the corpus albicans during the metestrus phase. Specifically, individuals carrying the deletion mutation exhibited smaller dominant follicles and corpus albicans. In healthy adult cows, larger follicles usually have a higher probability of being selected as dominant follicles during the follicular wave. Although this process is co-regulated by multiple factors, such as the luteinizing hormone surge [63], these results suggest a potential role for *CRY1* in ovarian dynamics. Furthermore, follicle size correlates with serum estradiol concentrations [64]. Following ovulation, the follicle transforms into the CL, which eventually undergoes luteolysis to form the corpus albicans [40]. While the corpus albicans is non-endocrine, its size reflects the regression status of the preceding CL [40]. Thus, the observed association suggests that *CRY1* may participate in follicular development and subsequent post-ovulatory structural changes. However, the relatively small sample size of the ID genotype subgroup, the lack of hormone and reproductive performance data and the lack of functional analysis may be considered as limitations of the current study.

### 4.4. Study Contribution and Implication

Despite these limitations, our findings provide valuable preliminary insights and generate testable hypotheses regarding the role of CRY1 in bovine fertility. Building upon previous reports. our study demonstrates a statistical link between CRY1 variants and ovarian morphological traits. These results suggest that CRY1 may have potential as a biological marker and as a candidate gene for improving bovine fertility. However, as an association study, these results establish correlation rather than direct causality; therefore, further large-scale functional studies are required to fully bridge the gap between CRY1 genotypes and localized ovarian physiology. The identified InDel, though requiring further validation, represents a potential candidate marker for future investigation, not an immediate tool for breeding programs. Marker-assisted selection (MAS) using variants in candidate genes offers a complementary approach to traditional phenotypic selection, particularly for traits with low heritability such as fertility. The high frequency of the II genotype in Holstein populations suggests that allele I may have been indirectly selected through its linkage with favorable production traits or may be maintained due to its potential beneficial effects on ovarian function.

## 5. Conclusions

A deletion mutation (P1-D6-bp) within the CRY1 gene showed a nominal association with the diameter of the dominant follicle and corpus albicans at metestrus in Chinese Holstein dairy cattle. These preliminary findings suggest that CRY1 may be associated with ovarian traits in cattle and could be a potential subject for further research into its role in reproductive function. However, these findings require validation in larger independent populations with appropriate correction for multiple testing. The current study may help to generate hypotheses for understanding the function of CRY1 and preliminarily explores its potential as a candidate marker for improving cattle fertility. Crucially, the biological basis for the estrus stage-specific observations, the influence of fixed effects such as season, age, parity, and source of ovary, and the lack of pedigree information are significant limitations that must be addressed in future investigations before any practical applications can be considered.

## Figures and Tables

**Table 1:** PCR primer pair sequences of the bovine *CRY1* gene.

Loci/Genes	Primer Sequences (5′ to 3′)	Location	Sizes (bp)	Variant ID
P1-D6-bp	F: CGTATGTGTTCTAACTCCTTCCCT R: ATCTAGACAGAAAAGACCCCAGT	Intron 1	254/248	rs133189221
P2-D5-bp	F: AAGCAGATGACACCTCCCTT R: TGGCTGTAGTCATCATCTGCA	Intron 1	231/226	rs442912813
P3-I8-bp	F: CCTCTTGGCTTTGCTGTTGT R: GCTCCAATACTTCAGCCACC	Intron 1	225/233	rs526350742
P4-D6-bp	F: TCCAATTTTACCCCATGTGCC R: CAGTGTTCTTGCCTGGAGAA	Intron 1	169/163	rs454970402
P5-D5-bp	F: CTTGTTACTTTGACAGCGAGGC R: TGTACACACTCTTGGGCTCAT	Intron 2	250/245	rs800313145
*CRY1-RT-qPCR*	F: CAGGAAGAGGACACGCAGAG	Exon	77	-
	R: ACCAAAGCTCTCCTCCTTACC			
*GAPDH-RT-qPCR*	F: CACTCACTCTTCTACCTT	Exon	91	-
	R: GCCAAATTCATTGTCGTA			

Note: “D” means Deletion, “I” means Insertion. In the “Sizes (bp)” column, the length in front of the “/” represents the non-mutated target fragment, and the length after the “/” represents the mutated fragment (insertion or deletion).

**Table 2:** Population genetic parameters of the *CRY1* gene P1-D6-bp loci in Chinese Holstein dairy cattle.

Genotypic Frequencies			Allelic Frequencies		HWE *p* Value	Population Parameters			
II	ID	DD	I	D		Ho	He	Ne	PIC
0.837 (*n* = 854)	0.163 (*n* = 166)	- (-)	0.919	0.081	*p* < 0.05	0.851	0.150	1.176	0.138

Note: “-” denotes “no detection”. HWE, Hardy–Weinberg equilibrium; Ho, observed homozygosity; He, expected heterozygosity; Ne, effective allele numbers; PIC, polymorphism information content.

**Table 3:** *CRY1* gene P1-D6-bp loci allelic frequencies in other cattle breeds.

Cattle Breeds	Number of Samples	Reference Allele Frequency (I)	Alternate Allele Frequency (D)
Charolais cattle	54	1.000	0
Simmental cattle	40	0.950	0.050

**Table 4:** Association between the P1-D6-bp variation within the *CRY1* gene and the ovarian traits (metestrus) of Chinese Holstein dairy cattle (LSM ± SE).

Quantitative Traits		Genotypes		*p*-Values
		II	ID	
Ovary	length (mm)	42.5 ± 0.6 (*n* = 223)	40.0 ± 1.3 (*n* = 45)	0.086
	width (mm)	22.5 ± 0.5 (*n* = 223)	23.6 ± 1.0 (*n* = 45)	0.328
	height (mm)	28.5 ± 0.4 (*n* = 222)	28.3 ± 1.0 (*n* = 45)	0.906
	weight (g)	12.3 ± 0.3 (*n* = 223)	12.0 ± 0.6 (*n* = 45)	0.749
Corpus luteum	number	1.3 ± 0.4 (*n* = 218)	1.2 ± 0.1 (*n* = 45)	0.076
	diameter (mm)	15.4 ± 0.5 (*n* = 214)	17.8 ± 1.8 (*n* = 44)	0.109
Dominant follicle	number	1.4 ± 0.1 (*n* = 78)	1.4 ± 0.2 (*n* = 15)	0.906
	diameter (mm)	12.1 ± 0.6 (*n* = 78)	8.6 ± 1.4 (*n* = 15)	**0.027**
Corpus albicans	number	1.5 ±0.1 (*n* = 62)	1.8 ± 0.3 (*n* = 13)	0.160
	diameter (mm)	4.6 ± 0.3 (*n* = 60)	2.8 ± 0.3 (*n* = 12)	**0.022**

Note: Bold font indicates a significant difference (*p* < 0.05). Genotypes are omitted when the number of individuals was less than three.

## Data Availability

The data presented in this study are available on request from the corresponding author upon reasonable request.

## Abbreviations

The following abbreviations are used in this manuscript:

Indel	Insertion–deletion
*CRY1*	Cryptochrome gene 1
PCR	Polymerase chain reaction
He	Expected heterozygosity
Ho	Observed homozygosity
Ne	Effective allele number
PIC	Polymorphism information content
HWE	Hardy–Weinberg equilibrium
SCN	Suprachiasmatic nucleus
PCOS	Polycystic ovary syndrome
II	Insertion/Insertion
ID	Insertion/Deletion
DD	Deletion/Deletion

## Appendix A

**Table A1:** Association between the 6 bp variation within the *CRY1* gene and the ovarian traits (diestrus, proestrus, and estrus) of Chinese Holstein dairy cattle (LSM ± SE) (*p* > 0.05).

Quantitative Traits		Genotypes		*p*-Values
		II	ID	
Diestrus (Stage of the estrous cycle)				
Ovary	length (mm)	41.2 ± 0.5 (*n* = 424)	42.4 ± 0.9 (*n* = 73)	0.281
	width (mm)	23.6 ± 0.3 (*n* = 424)	23.5 ± 0.9 (*n* = 73)	0.868
	height (mm)	25.5 ± 0.4 (*n* = 424)	25.2 ± 0.7 (*n* = 73)	0.714
	weight (g)	11.3 ± 0.3 (*n* = 392)	11.0 ± 0.6 (*n* = 73)	0.760
Corpus luteum	Number	1.7 ± 0.1 (*n* = 425)	1.8 ± 0.2 (*n* = 74)	0.221
	diameter (mm)	10.4 ± 0.4 (*n* = 419)	10.0 ± 0.9 (*n* = 74)	0.619
dominant follicle	Number	1.3 ± 0.1 (*n* = 115)	1.2 ± 0.1 (*n* = 25)	0.713
	diameter (mm)	9.9 ± 0.5 (*n* = 115)	11.5 ± 1.3 (*n* = 24)	0.226
Corpus albicans	Number	1.6 ± 0.2 (*n* = 110)	1.6 ± 0.2 (*n* = 19)	0.926
	diameter (mm)	4.0 ± 0.3 (*n* = 108)	4.1 ± 0.7 (*n* = 19)	0.971
Ovary	length (mm)	38.6 ± 1.0 (*n* = 105)	40.2 ± 1.7 (*n* = 21)	0.481
	width (mm)	18.1 ± 0.4 (*n* = 105)	16.1 ± 1.3 (*n* = 21)	0.091
	height (mm)	21.7 ± 0.8 (*n* = 105)	21.0 ± 2.0 (*n* = 21)	0.723
	weight (g)	7.0 ± 0.2 (*n* = 106)	6.7 ± 0.6 (*n* = 21)	0.520
Proestrus (Stage of the estrous cycle)				
Corpus albicans	number	1.4 ± 0.1 (*n* = 53)	1.7 ± 0.3 (*n* = 7)	0.236
	diameter (mm)	4.5 ± 0.4 (*n* = 53)	4.4 ± 0.6 (*n* = 7)	0.962
Estrus (Stage of the estrous cycle)				
Ovary	length (mm)	37.8 ± 0.8 (*n* = 93)	38.6 ± 1.8 (*n* = 22)	0.680
	width (mm)	18.8 ± 0.6 (*n* = 93)	19.3 ± 1.1 (*n* = 22)	0.734
	height (mm)	21.9 ± 0.6 (*n* = 93)	22.6 ± 1.4 (*n* = 22)	0.600
	weight (g)	7.8 ± 0.3 (*n* = 91)	8.2 ± 0.5 (*n* = 22)	0.456
dominant follicle	number	1.3 ± 0.1 (*n* = 93)	1.3 ± 0.1 (*n* = 22)	0.971
	diameter (mm)	11.8 ± 0.6 (*n* = 93)	11.5 ± 1.3 (*n* = 21)	0.801
Corpus albicans	number	1.3 ± 0.1 (*n* = 21)	1.5 ± 0.3 (*n* = 6)	0.570
	diameter (mm)	6.1 ± 1.0 (*n* = 21)	3.8 ± 0.6 (*n* = 6)	0.214

Note: Genotypes are omitted if the number of individuals is less than three.
